# Human papillomavirus-mediated carcinogenesis and HPV-associated oral and oropharyngeal squamous cell carcinoma. Part 1: Human papillomavirus-mediated carcinogenesis

**DOI:** 10.1186/1746-160X-6-14

**Published:** 2010-07-15

**Authors:** Liviu Feller, Neil H Wood, Razia AG Khammissa, Johan Lemmer

**Affiliations:** 1 Department of Periodontology and Oral Medicine, University of Limpopo, Medunsa Campus, South Africa

## Abstract

High-risk human papillomavirus (HPV) E6 and E7 oncoproteins are essential factors for HPV-induced carcinogenesis, and for the maintenance of the consequent neoplastic growth. Cellular transformation is achieved by complex interaction of these oncogenes with several cellular factors of cell cycle regulation including p53, Rb, cyclin-CDK complexes, p21 and p27. Both persistent infection with high-risk HPV genotypes and immune dysregulation are associated with increased risk of HPV-induced squamous cell carcinoma.

## Introduction

Cancer is a disease primarily caused by cytogenetic changes that progress through a series of sequential somatic mutations in specific genes resulting in uncontrolled cellular proliferation [1,2]. It may be caused by exposure to any one or more of a variety of chemical or physical agents, by random errors of genetic replication, or by errors in DNA repair processes. Almost all cancers follow carcinogenic events in a single cell (are monoclonal in origin), and this characteristic distinguishes neoplasms from hyperplasias that have a polyclonal origin [1].

Mutations in genes controlling cell cycle progression (gatekeeper genes) and DNA repair pathways (caretaker genes) are the essential initiating events of cancer. Both oncogenes and tumour suppressor genes act as gatekeeper genes. After mutation, certain genes may acquire new functions that lead to increased cell proliferation: these genes are called oncogenes. Such a mutational event occurs characteristically in a single allele of the future oncogene, and that allele then directly causes dysregulation of molecular mechanisms that control cell cycle progression. Tumour suppressor genes on the other hand, lose their function when both alleles are inactivated, and consequently lose their capacity to inhibit cell proliferation [1-7].

Caretaker genes are DNA repair-genes that serve to maintain the integrity and stability of the genome. Mutations in these genes do not directly contribute to uncontrolled cell proliferation, but increase the likelihood of mutations in the gatekeeper genes and may thus indirectly promote malignant cellular transformation [1,4,5,7].

Epigenetic modification refers to changes in gene expression (phenotype) without alteration in DNA structure (genotype). Somatic alterations of specific genes together with epigenetic events determine the development of malignancy. Significant among the epigenetic events are methylation of cytosine bases of DNA and modification of histones by acetylation or methylation which are associated with silencing of tumour suppressor genes [1-3,8-11].

Carcinogenesis can be seen as a Darwinian process involving sequential mutations giving the mutated cells growth dominance over the normal neighbouring cells resulting in the increased representation of the mutated cells in the affected tissue [12-15]. It is generally assumed that five to ten mutational events in as many different genes will transform a normal cell into a malignant phenotype [1,2].

The role of human papillomavirus (HPV) in the cellular bio-pathological processes of carcinogenesis of the anogenital region has been extensively researched and documented, and therefore Part 1 of this review is substantially based on this material. These bio-pathological sequential events are described in some detail as a basis for a discussion in Part 2 of the role of HPV in the pathogenesis of oral and oropharyngeal squamous cell carcinoma.

## Human papillomavirus (HPV)-induced carcinogenesis

High-risk HPV E6 and E7 oncoproteins expressed in epithelial cells infected with HPV are implicated in the increased proliferation and in the abnormal differentiation of these cells [16,17]. When the E6/E7 proteins are the expression of infection of the cell with low-risk HPV, then these active proteins may induce benign neoplasms. However, when E6/E7 proteins are the expression of high-risk HPV infection, they subserve the role of oncoproteins and they have the capacity to induce dysplastic and malignant epithelial lesions [18,19].

The association between cancer of the uterine cervix and high-risk HPV infection is well established. It is evident that HPV is an essential agent, but is not by itself sufficient to induce squamous cell carcinoma of the cervix. HPV DNA is found in more than 99% of biopsy specimens of squamous cell carcinoma of the cervix. In more than 70% of these HPV DNA positive biopsy specimens, the DNA is of high-risk HPV-16 and HPV-18 origin [20].

The prevalence of HPV infection of the cervix of the uterus is high, but in these same subjects the incidence of squamous cell carcinoma of the cervix is relatively low [21]. Therefore, besides persistence of the HPV infection, the HPV genotype, infection with multiple HPV genotypes, whether the viral DNA is present episomally or integrated and the quantum of cellular viral load may be important factors in the development of the cancer. Equally important may be other co-factors that may vary from individual to individual but can include immune fitness, nutritional status, the use of tobacco, and co-infection with other sexually transmitted agents including HIV and herpes simplex virus [20].

E6 and E7 oncoproteins can inactivate the genetic mechanisms that control both the cell cycle and apoptosis [16,17]. The hallmark of high-risk HPV E6 oncogenic activity is degradation of the p53 tumour-suppressor gene. The functions of p53 in the cell cycle include controlling the G1 transition to the S phase of the cell cycle at the G1 checkpoint by inducing expression of cyclin inhibitors p16, p21 and p27 that block the activities of cyclin-CDKs (cyclin-dependant kinase) complexes, thus mediating arrest of the cell cycle by blocking the progression of the cell cycle at the G1/S transition [17].

p53 activities mediate cell proliferation in response to mitogenic stimulation; mediate arrest of the cell cycle growth at the G1 checkpoint following DNA damage, hence permitting repair of the damaged DNA before the cell enters the DNA synthesis phase; and mediate induction of apoptosis of cells in which the DNA damage is beyond repair [22,23]. Therefore, inactivation, degradation, or mutation of the p53 gene may dysregulate its functions resulting in increased cell proliferation, in accumulation of damaged DNA, in growth of cells harbouring DNA errors, and in prolonged cell survival. However, loss of p53 function alone is not sufficient for the development of cancer, and other cytogenetic alterations are required for complete malignant transformation [22,23].

In addition to these properties, E6 oncoprotein of high-risk HPV types can also mediate cell proliferation through the PDZ-ligand domain [16]. The PDZ domain is located at areas of cell-to-cell contact, such as tight junctions of epithelial cells, and is associated with signal transduction pathways. The binding of high-risk HPV E6 oncoprotein to the PDZ family of proteins may result in degradation of the PDZ domain [24,25] leading to dysregulation of organization, differentiation, and of the chromosomal integrity of HPV infected epithelial cells [18]. This may contribute to morphological transformation of keratinocytes infected with high-risk HPV [26] and to induction of epithelial hyperplasia [27].

Telomerase is an enzyme that adds hexanucleotide repeats onto the end of the chromosome telomere [3]. Telomerase activity is usually restricted to embryonic cells and is absent in normal somatic cells [25]. When telomerase is absent, there is progressive shortening of telomeres as the cells repetitively divide, ultimately resulting in senescence of these cells [3,25,28]. HPV-induced activation of telomerase prevents the shortening of telomeres resulting in prolongation of the lifespan of HPV-infected cells [24,25,28].

High risk HPV E7 oncoprotein has the capacity to initiate DNA synthesis in differentiated epithelial cells mainly by binding and inactivating the Rb apoptosis/tumour-suppressor gene. The Rb family of proteins plays an essential role in controlling the cell cycle by governing the checkpoint between the G1 and the S phase. Hypophosphorylated Rb binds to E2F transcription factor forming a Rb-E2F complex, making E2F unavailable for transcription of genes associated with DNA synthesis. Upon phosphorylation of Rb by cyclin-CDK complexes, E2F is released from the Rb-E2F transcription repressor complex, and it induces transcription of the S-phase genes [16,18,23,25,29].

E7 oncoprotein of high-risk HPV types functionally inactivates the Rb family of proteins resulting in overexpression of E2F transcription factor with upregulation of cell cycle genes resulting in DNA replication, in the transition of the cell from the G1 to the S phase, and in increased cell proliferation [16,18,25].

E7 oncoprotein can also interact with other cellular factors that control the cell cycle including histone deacetylases, AP-1 transcription complex and CDK inhibitors, p21 and p27 [16]. Furthermore, E7 of high-risk HPV-16 and -18 can decrease the expression of major histocompatibility complex (MHC) class I molecules, thus interfering with MHC class I antigen presentation, resulting in downregulation of cellular immune responses, allowing HPV to persist in infected epithelial cells [17].

In addition to these properties, high-risk HPV E7 oncoprotein can induce chromosome duplication errors leading to dysregulation of mitotic spindle formation and function, contributing to the genomic instability of the cell [30].

The separate pathological effects of high-risk HPV E6 and E7 on the cell cycle complement each other, and together E6 and E7 mediate the HPV-associated epithelial cell transformation, and promote cellular genomic instability that predisposes the infected cells to full malignant transformation. High-risk HPV E7 activates the DNA synthesis and cell replication mechanisms that are normally inactive in matured epithelial cells, thus initiating pathological cell growth. By inducing cell survival and delayed apoptosis of cells with DNA damage, E6 allows E7 to exert and sustain its pathological effect [18].

Typically, infected epithelial cells of HPV-associated benign lesions harbour low-risk HPV episomally in the nuclei. In HPV-associated malignancies, high-risk HPV DNA may either be integrated within the cellular genome, or it may be maintained as an episome in the nuclei of the malignant cells [31]. It is unclear how the HPV genome, whether episomal within the nucleus or integrated into the nuclear cellular genome, brings about the same end result of malignancy [32].

**Figure 1:**
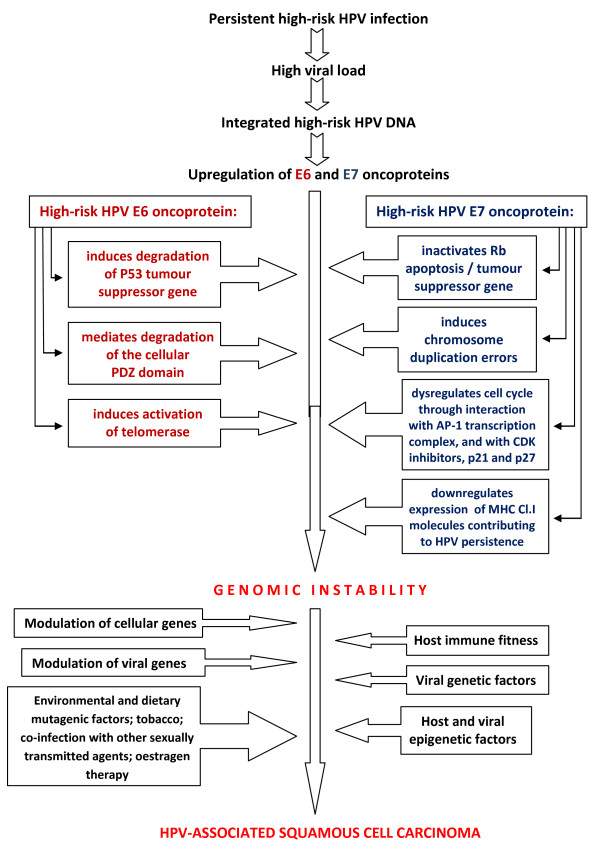
**Flow chart of high-risk HPV pathogenesis of squamous cell carcinoma**. By inactivation of p53, high-risk HPV E6 oncoprotein induces cell survival and delayed apoptosis, and HPV E7 oncoprotein through inactivation of Rb gene stimulates cellular DNA synthesis and pathological cell growth. The separate pathological activities of HPV E6 and E7 on the cell cycle complement each other and mediate the HPV-associated epithelial cell transformation.

The integration of HPV DNA favours the inactivation of tumour suppressor genes, p53 and Rb, contributing to increased cellular chromosomal instability, and prolonging the lifespan of the cell, essential steps in the multi-step process of HPV-associated carcinogenesis [11,25,28,33]. It is probable that following the initial HPV-induced cellular transformation, additional interactions with chemical carcinogens will provide the necessary additional impetus for the development of frank malignancy (Figure 1) [32].

The integration of the HPV genome as opposed to the presence of HPV episomally is associated with a greater frequency of cervical intraepithelial neoplasia (CIN) grade 3, and with invasive squamous cell carcinoma of the uterine cervix [11,28,34]. The pathological significance of integration is not entirely clear since HPV often exists concurrently in both episomal and integrated forms. The chromosomal locations of integrated HPV are very variable, and there is a paucity of data on the frequencies and chromosomal locations of different HPV genotypes [11,35].

HPV oncoproteins can act synergistically with intra-nuclear proto-oncogenes, with cytokines that bind and activate E6/E7 promoter, with exogenous factors including carcinogens in tobacco and dietary agents, steroids, and UV and X-radiation, to promote HPV-tumourigenesis (Figure 1) [31].

## Genetic and epigenetic events associated with HPV infection

The cellular genomic integrity is maintained by various caretaker cellular systems, including DNA monitoring and repair enzymes, checkpoints that regulate the cell cycle, and genes that ensure the accurate chromosomal replication during mitosis. Malfunction of cellular caretaker systems brings about genomic instability that is associated with increased risk of acquiring accumulative genetic alterations that can ultimately culminate in carcinogenesis. The genomic instability brought about by HPV-induced malfunction of p53 tumour suppressor gene results in the inheritance of abnormal DNA by cells that are not only proliferating in increased numbers, but surviving longer with consequently increased chances of malignant transformation [3].

Tumours destined to become malignant appear to be characterized by chromosomal imbalances, in terms of gains or losses of genetic material [36]. Most chromosomal imbalances affect large genomic regions containing multiple genes, and have functional consequences that are unknown. Gains or losses of genetic material lead to changes in DNA copy-numbers [37]. Genomic gain may arise from DNA sequence amplification leading to overexpression of oncogene products; and genomic losses may be brought about by single-gene or intragenic deletion leading to the loss of the functional product of a tumour suppressor gene [1,36].

Large-scale genomic gains or losses affecting multiple genes are frequently observed in cancers and manifest in changes in DNA copy-numbers, but the identification of the specific gained or lost gene that promotes the carcinogenesis is difficult, and in most cases impossible [36].

HPV-related anal intraepithelial neoplasia is associated with DNA copy-number abnormalities, and the severity of the lesion is directly related to the magnitude of the DNA copy-number changes [33].

In HPV-induced malignancies there are two distinct epigenetic events. The first is methylation of viral genes that are associated with increasing viral oncogenic capacity, and the second is silencing of cellular tumour-suppressor genes through hypermethylation of the promoter regions [11]. Given enough time, the accumulation of epigenetic and genetic changes may eventually cause malignant transformation [33].

## Conclusions

As is the case in many other malignancies, HPV-induced carcinogenesis is a complex process characterized by alterations in genes encoding tumour-suppressor genes and by epigenetic modifications. The hallmark of HPV-induced carcinogenesis is inactivation of p53 tumour-suppressor gene by the E6 and of Rb apoptosis/tumour suppressor gene by E7 oncoproteins of high-risk HPV genotypes. The aberrant function of these genes and the consequent genomic instability compounded by the additive effects of one or more cofactors leads to preferential growth of the affected cells which characterize the progressive uncontrolled growth in cancer.

## Competing interests

The authors declare that they have no competing interests.

## Authors' contributions

LF and RAGK contributed to the literature review. LF, JL and NHW contributed to the conception of the article. LF, JL, NHW and RAG contributed to the manuscript preparation. Each author reviewed the paper for content and contributed to the manuscript. All authors read and approved the final manuscript.
